# Food Safety: Allergen Labeling Takes Effect

**DOI:** 10.1289/ehp.114-a24

**Published:** 2006-01

**Authors:** Richard Dahl

Since 1994 food manufacturers have been required to list all the ingredients on their products’ labels. A new law now takes this obligation a step further, requiring manufacturers to notify consumers in “plain language” of certain allergens contained in their products. This is good news for the estimated 11 million Americans who have food allergies. But some question whether the new labels might be too much of a good thing.

The Food Allergen Labeling and Consumer Protection Act of 2004, or FALCPA, applies to foods labeled on or after 1 January 2006. It mandates that the nutritional labels on food packages plainly identify any of eight specified food allergen sources— milk, eggs, fish, crustacean shellfish, tree nuts, peanuts, wheat, and soybeans— that are present in the product. Together, these eight food categories account for about 90% of all food allergies. The law stipulates that the warning label be placed near the ingredient list.

Stephen L. Taylor, who heads the Food Processing Center at the University of Nebraska–Lincoln, lauds the “plain language” requirement as an overdue development. “In the past, you’ve seen terms like ‘casein’ and ‘whey,’” he says. “Consumers often had to learn the hard way that those terms are synonymous with ‘milk.’”

But while the new law makes the presence of certain allergens in food products more understandable, Taylor also contends that the act is too strict in requiring that allergens be listed if they are present in the faintest traces. For example, he says, the law requires the listing of not only ingredients but also processing aids that may include allergens, such as soybean lecithin, which is used by baking companies as a stick-release agent for pans.

“My view is that in this particular application the exposure to soybean allergens is extremely low, but with the new labeling requirements you’re going to be advising all soy-allergic individuals not to eat the vast majority of bakery products,” Taylor says. “And I don’t think that’s particularly in their best interests.”

The law makes clear that decisions about allergen labeling for food products will be an ongoing process. It requires that the Secretary of Health and Human Services provide a report to Congress in February 2006 that’s to include information about unintentional contamination of foods with allergens stemming from equipment that is used for multiple food processes. In addition, the U.S. Food and Drug Administration has created the Threshold Working Group to examine approaches that could be used to establish thresholds below which manufacturers would not be required to list food allergens.

Anne Muñoz-Furlong, founder and chief executive officer of the Food Allergy & Anaphylaxis Network (FAAN), a nonprofit educational organization, considers the law an important step. “With food allergies, there’s no cure,” she explains. “[Allergic] individuals depend on other people, whether in a restaurant or the food industry, to provide accurate information so they can make the right choices.”

According to figures from FAAN, each year some 30,000 Americans require emergency room treatment for allergic reactions to food, and 150 to 200 people die from such reactions. Furthermore, the number of people with food allergies is increasing around the world.

Of particular concern to many food allergists is the sharp increase of food allergies in children. According to A. Wesley Burks, a professor of pediatrics at Duke University Medical Center, peanut allergies have doubled over the last decade among children under the age of five.

Nobody really knows why allergies are on the rise. One theory holds that improved hygiene leaves the human immune system with less to do, Muñoz-Furlong says, so it identifies a particular food as dangerous and responds by attacking it.

Muñoz-Furlong believes that the next step in the development of allergen labeling should be to create binding guidelines for what is currently the voluntary use of “precautionary labeling,” which warns of the possibility that an allergen might be present as the result of shared production processes. As for the longer-term issue of how to establish threshold levels, Muñoz-Furlong says that most of the parents of food-allergic children she’s talked to believe the answer is simple: “They want zero. They don’t want to risk that their child might be in that small percentage of the population that’s below the threshold.”

## Figures and Tables

**Figure:**
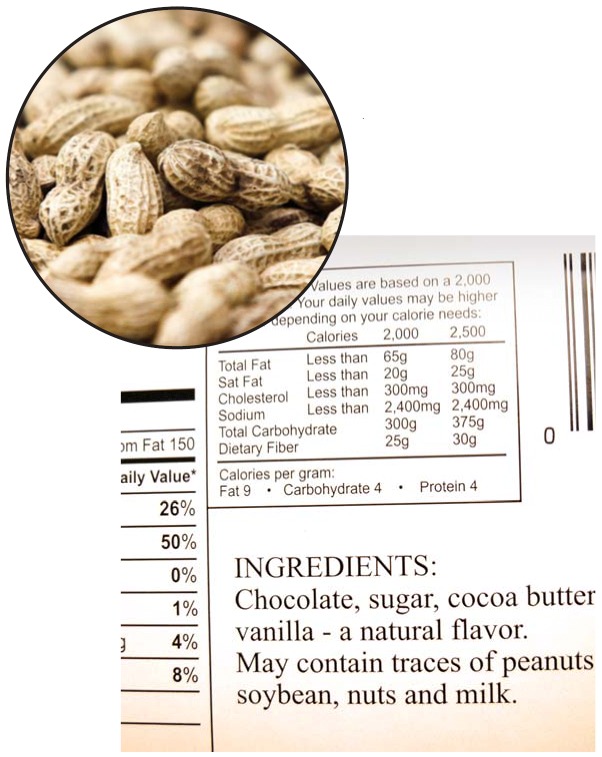
Plain talk about allergens. New labeling requirements should make it easier for allergic consumers to tell if a food is safe for them to eat. Next up? Some suggest codifying the now-voluntary use of precautionary labeling (large photo).

